# Interrelation between Fiber–Matrix Interphasial Phenomena and Flexural Stress Relaxation Behavior of a Glass Fiber–Polymer Composite

**DOI:** 10.3390/polym13060978

**Published:** 2021-03-23

**Authors:** George C. Papanicolaou, Diana V. Portan, Lykourgos C. Kontaxis

**Affiliations:** Composite Materials Group, Department of Mechanical Engineering and Aeronautics, University of Patras, 26500 Patras, Greece; diana.portan@gmail.com (D.V.P.); lykourgoskontaxis@gmail.com (L.C.K.)

**Keywords:** interphase, modeling, viscoelasticity, flexural stress relaxation, fiber–matrix adhesion, fiber volume fraction, time dependence

## Abstract

The response of fiber-reinforced polymer composites to an externally applied mechanical excitation is closely related to the microscopic stress transfer mechanisms taking place in the fiber–matrix interphasial region. In particular, in the case of viscoelastic responses, these mechanisms are time dependent. Defining the interphase thickness as the maximum radial distance from the fiber surface where a specific matrix property is affected by the fiber presence, it is important to study its variation with time. In the present investigation, the stress relaxation behavior of a glass fiber-reinforced polymer (GFRP) under flexural conditions was studied. Next, applying the hybrid viscoelastic interphase model (HVIM), developed by the first author, the interphase modulus and interphase thickness were both evaluated, and their variation with time during the stress relaxation test was plotted. It was found that the interphase modulus decreases with the radial distance, being always higher than the bulk matrix modulus. In addition, the interphase thickness increases with time, showing that during stress relaxation, fiber–matrix debonding takes place. Finally, the effect of fiber interaction on the interphase modulus was found. It is observed that fiber interaction depends on both the fiber–matrix degree of adhesion as well as the fiber volume fraction and the time-dependent interphase modulus.

## 1. Introduction

Structural engineers have for a long time adopted fiber-reinforced polymer composites (FRPs) as basic materials to construct different advanced engineering structures, with their usage ranging from aeronautics and marine structures through to automobiles, sports goods and civil infrastructure such as bridges and buildings [1,2,3]. Nowadays, FRP usage continues to grow at an impressive rate, creating new markets such as biomedical-specific applications and devices [4,5]. In addition, the development of carbon nanotubes, nanofibers and nanoparticles and their usage as polymer reinforcements have rendered polymer composites promising structural engineering materials for diverse present and future applications [6].

Depending on the nature of the constituent phases and the type of interaction between them, the fiber–matrix interphase area is an area of a complicated structure characterized by microcracks, impurities, reduced polymer molecules mobility due to polymer matrix molecules’ adsorption onto the fiber surface, voids, etc. As a result, the fiber–matrix interphase in a composite material is a phase with totally different properties compared to the matrix and the reinforcement, acquiring properties from both constituent phases in contact and having a tremendous impact on the final composite’s macroscopic mechanical and viscoelastic properties [7,8]. Fiber and matrix concentrations affect the structure and the property-dependent interphase extent, which, in turn, affects the overall behavior of the composite. As a result, even a slight change in the volume fraction of the reinforcement may lead to significant changes in the properties of the final polymer composite [9,10,11,12]. The interphases in fiber-reinforced polymers need to be widely investigated since it is acknowledged that these areas have a pronounced effect on the bulk properties of composite materials [13], especially under extreme loadings and harsh environmental conditions [14,15,16,17]. Two important parameters that shall be considered and intercorrelated for a deeper understanding of fiber-reinforced composites’ mechanical response to different loading modes are the reinforcing volume fraction and the quality of adhesion between the fiber and matrix. Previously reported studies declare that strain decreases with an increase in fiber content. Consequently, the relationship between composite mechanical properties and fiber volume fraction should be intensively studied for various composites from both experimental and theoretical viewpoints [18].

Models have always been acknowledged as an appreciated attempt to decode the real world to the best of our knowledge. Modeling and optimization can take many forms, covering a combination of operating variables, depending on the technological need being addressed [19,20,21,22,23]. The benefits of models that are used to predict the behavior of materials under different environmental and loading conditions are many; these are related to the selection of appropriate materials, the reduction of the experimentation cost and shortening the time needed to standardize a material and release it on the market. Models can be accustomed to the type of material, at different scales and for specific applications, depending on the industrial sector involved (aerospace, automotive, marine, etc.). Through the prediction of the final composite’s properties, materials can be combined to obtain ideal characteristics suitable in engineering applications, thus avoiding the trial-and-error method and also maximizing high structural performance and a sustainable safe life [24,25,26,27,28].

Previous efforts have been made to study and clarify the effect of several parameters such as aspect ratio, fiber volume fraction, fiber–matrix modulus ratio, fiber spacing and fiber end-gap size influencing the mechanical properties (damping behavior) of composite and complex structures [29]. Despite all previous efforts made by several researchers, there are not any investigations in literature, until now, studying the effect of several structural parameters such as fiber–matrix adhesion, fiber volume fraction, interphase modulus and interphase thickness on the overall viscoelastic behavior (stress relaxation) of FRPs. The present investigation is a novelty since it is an effort to find the interrelation existing between all the above-mentioned parameters. More precisely, the aim of the present study is to investigate the stress relaxation behavior of a glass fiber-reinforced polymer (GFRP) and predict its fiber–matrix interphase modulus, thickness and time dependence using the hybrid viscoelastic interphase model (HVIM), which is a semiempirical model developed by Papanicolaou [30]. In this model, values of the relaxation modulus are correlated to the interphase thickness, and the fiber volume fraction and fiber–matrix adhesion quality are interplayed to alter the overall behavior of the composite and predict its limits when in service.

## 2. Materials and Methods

### 2.1. Materials

The material tested was a glass-fiber–polymer matrix composite, supplied in the form of plates acquired by R&G GmbH (Waldenbuch, Germany). It corresponds to NEMA-grade FR-4 and meets the requirements of IPC-4104C/21. The GFRP-plate was 2 mm in thickness, having 40 layers of 80 gm glass fabric, with a layer thickness of 0.05 mm in a 0°/90° fiber orientation. The fiber volume fraction was 60%. The main mechanical properties of the GFRP material are presented in Table 1.

The epoxy system used as matrix material was RenLam CY219 (Bisphenol A) resin combined with an HY 5161 (amine) curing agent at a ratio of 2:1 by weight. Gel time was 24 h at 50 °C, and the density of the cured polymer was 1.16 g cm^–3^. The viscosity of the system was 1–1.2 Pas at 25 °C.

### 2.2. Quasistatic Mechanical Tests

**Figure 1:**
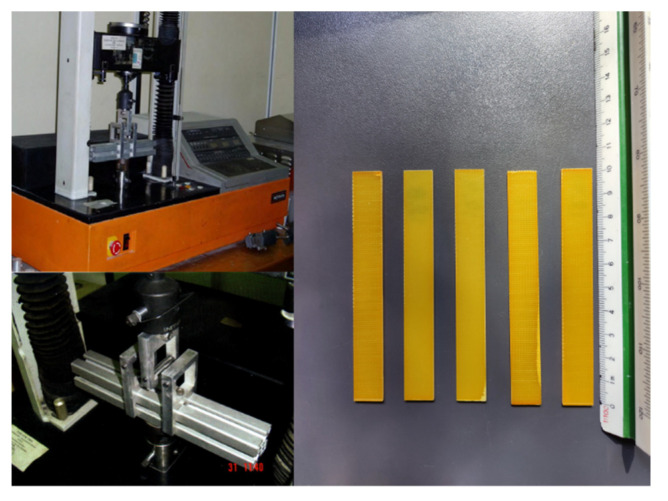
Experimental setup and glass fiber-reinforced specimens.

Pure epoxy resin specimens and GFRP composites underwent a series of quasistatic three-point bending experiments (ASTM D790-03) using an Instron 4301 (High Wycombe, UK) universal mechanical testing machine. The tests were performed at room temperature to investigate the mechanical properties of the composites. In all cases, a constant crosshead speed of 1 mm/min was applied. All specimens had dimensions of 100 × 12.8 × 2 mm and a span length of 63 mm (Figure 1). Five or more specimens per each case (i.e., pure resin, GFRP) were tested to ensure the repeatability of results.

### 2.3. Stress Relaxation Tests

Pure epoxy resin and GFRP composite specimens had dimensions according to ASTM D790-03 standards. Stress relaxation experiments were executed using an Instron 4301 (High Wycombe, United Kingdom) universal testing machine. The specimens had dimensions of 100 × 12.8 × 2 mm and were placed on support rollers with a span of 63 mm (Figure 1). Initially, the machine started and continued to operate with a constant crosshead speed of 1 mm/min until the desirable displacement was reached. Then, the machine was stopped at the desirable displacements (4, 5 and 6 mm) for 45 and 100 min for the neat epoxy resin and composite specimens, respectively. Five or more specimens per each applied displacement value were tested to ensure the repeatability of results.

## 3. Theoretical Background

One of the major disputes of stiff fibers embedded in a soft epoxy matrix is related to the existence of interfaces separating constituent material phases. In a wide range of technological applications, interfaces extend to interphases that withstand large-enough thicknesses to significantly affect the overall material properties. According to the IUPAC Compendium of Chemical Terminology, “*an interfacial layer is defined as the inhomogeneous space region intermediate between two bulk phases in contact, and where structural characteristics and properties are significantly different from, but related to those of the bulk phases*.” Examples of such parameters are composition, molecular density, orientation or conformation, charge density, stress tensor, etc. Within the interphase, properties vary in the direction normal to the surface of the fiber, with a pronounced or smooth jump depending on the fiber–matrix adhesion efficiency. Coexisting sorption or depletion regions of one or several components end up in the formation of complex profiles of interphase properties. In the sequence, the progress of the development and application of the HVIM is presented.

### 3.1. Interphase Model

The interphase model was the first form of the HVIM, developed to describe the interphase quality and its influence on the behavior of different mechanical systems. The most decisive factor that impacts the interphase quality was found to be related to the adhesion efficiency at the interface between two phases. At that time, in most theoretical models, the adhesion was considered perfect so that it could ensure the continuity of stresses and displacements. However, such a condition is hardly fulfilled in real composites. Moreover, something much more complicated than a simple mechanical effect occurs because of the filler–matrix interaction. An avalanche of chained events starts within the composite and affects firstly its structure and ultimately its overall properties. This is triggered by the reduced or increased mobility of the polymer molecules around the reinforcement, a phenomenon that was already mentioned in the introduction of the present work.

The interphase model was developed in the 1980s by Papanicolaou with the purpose of more accurately predicting the real nature of the interaction between the reinforcement and matrix material [32]. This model consisted of three concentric cylinders, each one representing the filler, the interphase and the matrix material, respectively [33]. It was considered that all interphase properties varied within the same interphase thickness in a direction normal to the fiber surface. Improvement of this model was later achieved and is presented in the sequence.

### 3.2. Hybrid Interphase Model

As previously mentioned, a percentage of the bulk matrix surrounding the inclusions in a fiber-reinforced composite suffers structure and composition modification according to the type of interaction of the initial phases when coming into contact. Due to the technological progress of microscopy, these regions were easily detected, and in some cases, their thickness could be measured. Based on this progress, in a series of recent investigations, a novel approach to the interphase concept was introduced. Focusing on one specific property within the interphase, the thickness of this region is calculated according to this property and is defined as the maximum radial distance from the inclusion boundary at which the property in discussion varies. In this area, the bulk matrix is strongly affected, and the interphase volume fraction is defined as the percentage of the bulk matrix surrounding the inclusions in which a specific matrix property is strongly affected by the existence of the reinforcement. Unavoidably, the old interface concept, described as a line separating two phases, was considered totally inappropriate to model a composite’s behavior. The real interphase, having a property depended thickness, determines the formation of the volume fraction of the modified matrix surrounding the inclusion and is a complex structural concept. Given that most studies target the investigation of more than one property at the interphase, we discuss the so-called hybrid interphase, where each of the chosen properties determine the formation of a different interphase thickness. Based on this approach, the hybrid interphase model (HIM) was developed [27,34,35].

### 3.3. Hybrid Viscoelastic Interphase Model

A very important parameter that has not been included in the previously mentioned interphase models is time. Further improvement of the hybrid interphase model considered the fact that the value of a chosen property depends on the moment in time when this is measured. Involving the time in the equation, we may assume the existence of a nonhomogeneous viscoelastic and anisotropic interphase. Combining time and the effect of the degree of adhesion between phases, which is expressed as the abrupt jump in properties at the inclusion–interphase boundary, the situation becomes much more complex. Given that interfacial phenomena and the interphase region, in general, are critical for the composite’s global response, the viscoelastic behavior of the interphase was modeled by introducing the concept of hybrid viscoelastic interphase [30].

Because of the nature of the polymeric matrix, the viscoelastic behavior of the interphase is matrix dominated. It has been observed that the degree of adhesion between the constituent phases is greatly affected by the time instant of observation and has a strong impact on all properties of the hybrid interphase. Finally, it tremendously affects the overall viscoelastic response of fiber-reinforced composites. This improved form of the hybrid interphase model was named the hybrid viscoelastic interphase model and applies for any physical/mechanical property (e.g., mechanical, thermal, electrical and/or biomechanical) [36]. Knowing the interphasial variation of a specific property, one can predict the corresponding macroscopic behavior of the composite system.

### 3.4. Application of the Hybrid Viscoelastic Interphase Model

#### 3.4.1. Geometrical Aspects

**Figure 2:**
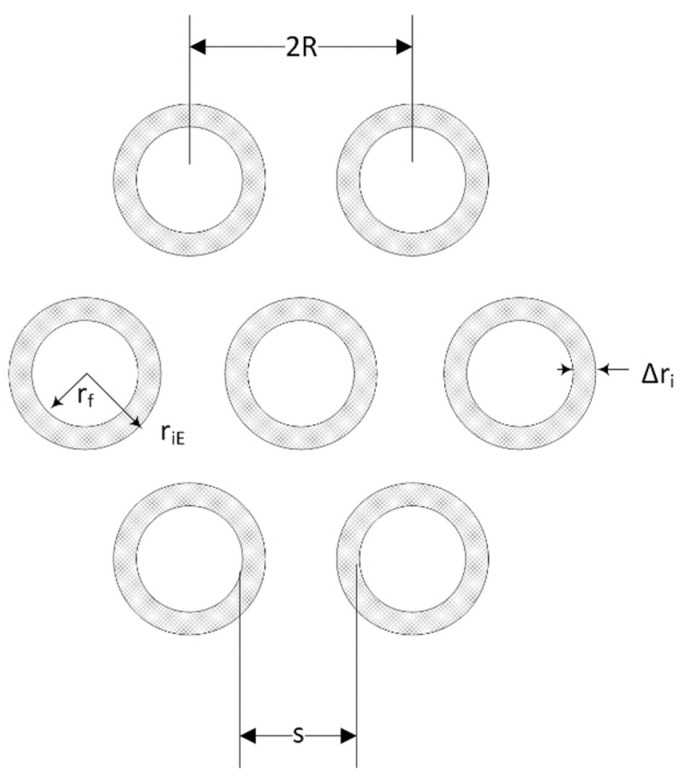
Hexagonal array of fibers in a unidirectional fiber composite. Fibers are surrounded by an interphase area.

In the present investigation, assuming that the fibers are arranged on a hexagonal lattice, each fiber has a circular cross-section of the same diameter, as shown in Figure 2.

For the ideal hexagonal arrangement, the volume fraction of fibers V_f_ is related to the fiber radius as:

(1) $$V_{f}=\frac{\pi }{2\sqrt{3}}\cdot {\left(\frac{r_{f}}{R}\right)}^{2}$$

Further, the separation of the fibers S equals:

(2) $$S=2\left(R-r_{f}\right)$$

From Equations (1) and (2) V_f_ is given as:

(3) $$V_{f}=\frac{\pi }{2\sqrt{3}}\cdot {\left(\frac{2r_{f}}{S+2r_{f}}\right)}^{2}$$

So that the fiber separation distance is given by:

(4) S=2[(π2Vf3)12−1]rf

#### 3.4.2. Model Application

As shown in [30], one of the main factors affecting the interphase thickness is the degree of the adhesion coefficient k_E_. This is defined as:

(5) $$k_{E}=\frac{E_{i}(r_{f}^{+},t)}{E_{f}}.$$

The lower the value of the adhesion coefficient, the more inefficient the bonding between the fiber and the matrix macromolecules. As the molecules of the matrix are weakly bonded to the fiber surface, the interphase thickness increases.

It is obvious that perfect bonding between fiber and matrix does not exist in real composites due to the existence of flaws and fiber surface roughness, as well as other physical and mechanical interactions. This imperfection is described by the adhesion coefficient, which represents the discontinuity of the properties that occur at the fiber–matrix interface. The coefficient k_E_ describes equivalently the efficiency of bonding for the modulus of elasticity, as defined by Equation (5). This equation imposes a jump on material properties and can take values 0 ≤ k_E_ ≤ 1. The limit value k_E_ = 1 describes a compliant interphase with perfect adhesion conditions, in which full stress transfer occurs. When its value is zero, no stress transfer occurs. In real conditions of imperfect adhesion, where 0 < k_E_ < 1, only a part of the stresses is transferred from the matrix to the fiber through the interphase such that the fiber volume fraction is replaced by the effective fiber volume fraction:

(6) $$V_{eff}=k_{E}V_{f}.$$

The above accounts for any material property such as Poisson’s ratio, the thermal expansion coefficient, etc.

Moreover, the mechanical properties in the transverse direction are dominated by the matrix and more precisely by the part of the matrix affected by the presence of the fiber or, in other words, the interphase extent. On the contrary, the composite longitudinal properties are principally dominated by the fiber. As a result, the interphase thickness should be proportional to the anisotropy coefficient of the material S_E_. The value of a property in the longitudinal direction is proportional to the effective fiber volume fraction. The corresponding equations are given below:

(7) $$E_{L}=E_{f}V_{eff}+E_{m}(1-V_{eff}),$$

(8) $$E_{T}=\frac{E_{f}E_{m}}{E_{m}V_{eff}+E_{f}(1-V_{eff})},$$

(9) $$S_{E}=\frac{E_{T}}{E_{L}}$$

The interphase thickness was calculated according to the following relation [30]:

(10) $$\Delta_{ri}=-\frac{(1-k_{E})\cdot S_{E}\cdot r_{f}}{k_{E}}\ln\left[\frac{10^{-3}}{k_{E}E_{f}-E_{m}\left(t\right)}\right].$$

In addition, the elastic modulus variation within the hybrid interphase region is given by:

(11) $$E_{i}(r,t)=E_{m}(t)+(k_{E}E_{f}-E_{m}(t))\cdot \exp\left\{-\frac{k_{E}}{1-k_{E}}\frac{E_{L}}{E_{T}}\frac{r-r_{f}}{r_{f}}\right\}\;\mathrm{with}\;r_{f}\leq r\leq r_{iE},$$

where E_f_ is the fiber modulus, E_i_ is the interphase modulus, E_m_(t) is the time-dependent matrix modulus, E_L_ and E_T_ are the macroscopic longitudinal and transverse moduli of the single fiber representative volume element (RVE), respectively, and k_E_ is the fiber–matrix adhesion coefficient with respect to the modulus.

## 4. Results and Discussion

### 4.1. Application of the HIM Model on the Quasistatic Bending Results

**Figure 3:**
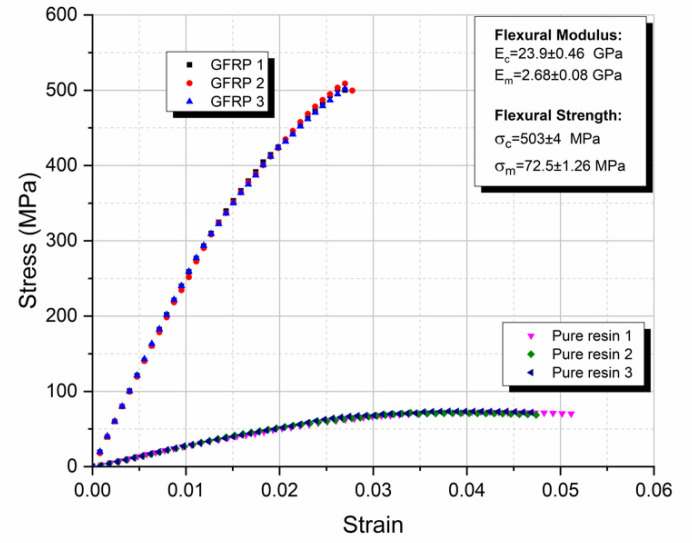
Three-point bending tests of the GFRP composites and the neat epoxy resin.

In Figure 3 the results of the quasistatic three-point bending tests are presented, for neat epoxy resin and GFRP composite specimens. Quasistatic three-point bending tests were conducted, to verify the material properties given by the manufacturers.

From this figure, the repeatability of experimental results can be observed. The flexural moduli of both materials are well within the manufacturers’ data, as shown in Table 1. The flexural strength of the GFRP composites and that of the neat epoxy resin was found equal to 503 ± 4 and 72.5 ± 1.26 MPa, respectively.

**Figure 4:**
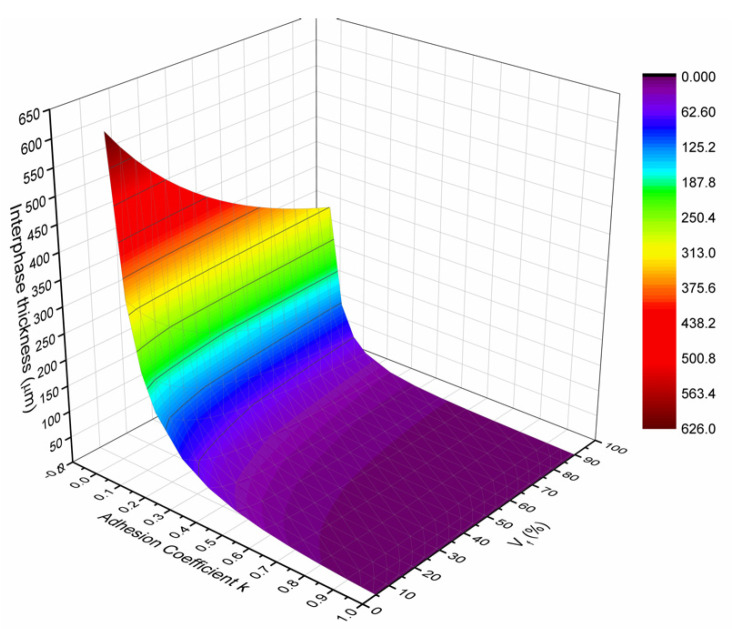
Prediction of the interphase thickness Δr_i_ as a fraction of the volume fraction V_f_ and the adhesion coefficient k_E_.

The HVIM was able to predict the interphase thickness, initially under quasistatic bending conditions. The two main parameters affecting the interphase thickness are the adhesion coefficient k_E_ and the fiber volume fraction V_f_. Therefore, a parametric study, using Equation (10), was conducted to study the interphase thickness dependence upon k_E_ and V_f_ (Figure 4).

As stated earlier, the most important factor affecting the interphase thickness is the adhesion coefficient k_E_. To better understand this, one must consider the interphase material structure. The interphase is a region around the fiber that, besides the anchored polymeric chains on the fiber surface, also consists of microcracks, cavities between the fiber and matrix, impurities and voids. The existence of these discontinuities leads to the reduction of the adhesion bond and consequently to a respective reduction of the amount of stresses transferred from the matrix to the fiber. Consequently, the lower the adhesion coefficient k_E_, the larger the interphase thickness; on the contrary, the higher the adhesion coefficient k_E_, the smaller the interphase thickness. This behavior is predicted by the HIM and is depicted in Figure 4, where the interphase thickness reduces exponentially as the adhesion coefficient increases.

**Figure 5:**
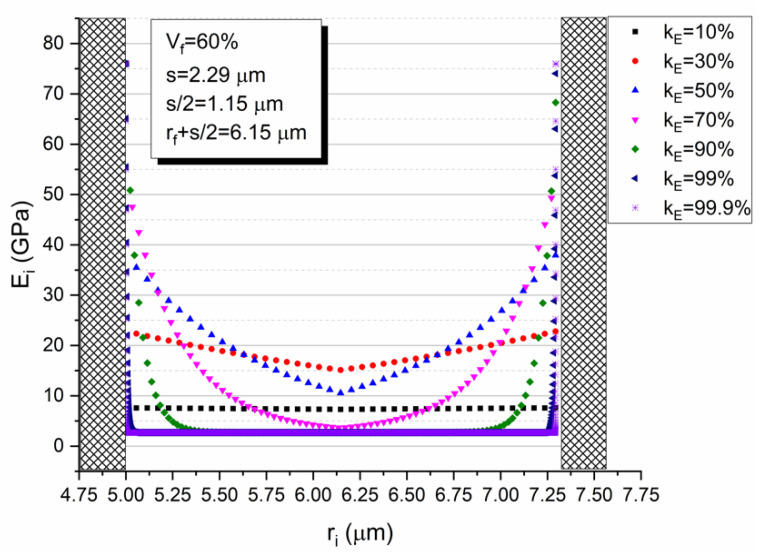
Interphase modulus variations within the interphasial area between two adjacent E-glass fibers for different values of the adhesion efficiency coefficient k_E_.

Although all tests were executed in specimens with a 60% fiber volume fraction, it is interesting to present the variation of the interphase thickness as a function of the fiber volume fraction V_f_, as derived by the HIM and shown in Figure 4. It can be observed that the interphase thickness decreases exponentially as the volume fraction increases. This is attributed to the fact that as V_f_ increases, the separation distance between the fibers decreases too so that at a certain volume fraction the separation distance becomes so small that the interphase areas of two successive fibers may overlap. Under such a condition, the whole matrix material is being modified having properties different than those of the neat resin. Therefore, a parametric study, using Equation (11), was conducted to study the interphase modulus dependence upon ri for different values of k_E_ (Figure 5).

As aforementioned, assuming a hexagonal array of fibers, the separation distance S, can be calculated from Equation (4). Thus, the interphase modulus variation, within the interphasial area between two adjacent E-glass fibers, for different values of the adhesion coefficient k_E_, can be plotted. From this diagram, it can be seen that for k_E_ < 0.70, the matrix material existing in the area between two successive fibers is totally modified, having a modulus higher than that of the neat matrix.

**Figure 6:**
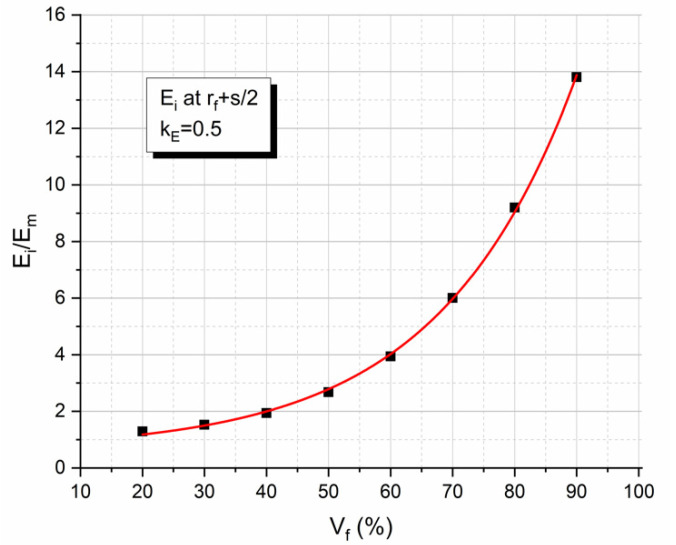
Variation of the relative interphase modulus E_i_/E_m_ value corresponding to the midpoint of the fiber separation distance between two adjacent E-glass fibers with the fiber volume fraction.

Furthermore, in Figure 6 the relative interphase modulus E_i_/E_m_ (see Equation (11)) is plotted against the fiber volume fraction V_f_ for the GFRP composite considered. At this point, it should be mentioned that for the composite under consideration, k_E_ was found to be equal to 0.5. From this diagram, it is clear that even the minimum value (corresponding to the midpoint of the fiber separation distance) of the relative interphase modulus is always higher than the modulus of the neat resin for all fiber volume fractions, while there is a continuous increase of this value with the filler volume fraction V_f_.

**Figure 7:**
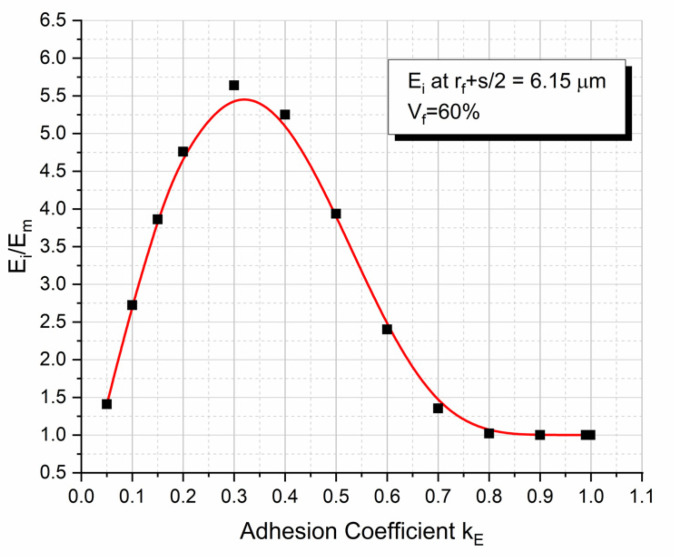
Normalized interphase-to-matrix modulus at the half-distance between two adjacent E-glass fibers for a 60% volume fraction for different adhesive coefficients k_E_.

However, when plotting the interphase relative modulus, E_i_/E_m_ value (see Equation (11)) corresponding to the midpoint of the fiber separation against the adhesion coefficient k_E_ (Figure 7), one can observe an initial and almost abrupt increase, reaching a maximum for k_E_ = 0.3 and this is followed by a subsequent reduction of the ratio E_i_/E_m_. This can be explained when simultaneously observing Figure 5 and Figure 7. At very low k_E_ values (0 < k < 0.3), as already mentioned above, the interphase extent for each of the two fibers is too high resulting in the overlapping of the two interphase areas. As k_E_ increases, the interphase extent decreases imposing mobility constraints to the matrix macromolecules in contact with the fiber surface. Thus, the relative interphase modulus increases. However, except for the interphase extent parameter, one must take into account two additional parameters; i.e., the maximum value of the relative interphase modulus at the fiber–matrix contact point, and the rate of decrease of the relative interphase modulus as the radial distance from the fiber surface increases. What is happening is that for k_E_ values greater than k_E_ = 0.3 in addition to the decrease in the interphase thickness, both the relative interphase modulus at the fiber–matrix contact point and the rate of decrease of the relative interphase modulus increase. As a result, the relative interphase modulus E_i_/E_m_ value at the midpoint of the fiber separation distance between two adjacent E-glass fibers decreases.

**Figure 8:**
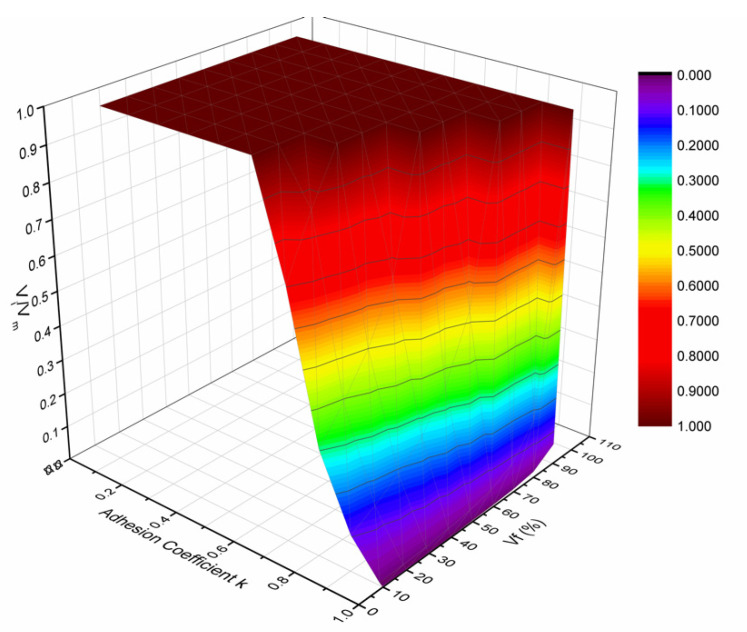
Three-Dimensional diagram showing the variation of the relative interphase volume fraction V_i_/V_m_ versus the adhesion coefficient k_E_ and the fiber volume fraction, V_f_.

All the above-presented variations are summarized in Figure 8 where a 3D diagram is plotted showing the variation of the relative interphase volume fraction (V_i_/V_m_ = V_i_/(1 − V_f_)) versus the adhesion coefficient k_E_ and the fiber volume fraction, V_f_. The resulting diagram is characteristic for glass fiber–epoxy composites with constituent properties as given in Table 1. The above 3D diagram shows that for low k-values, V_i_ = V_m_ means that all matrix material is modified into the interphase material. As the k-value increases, i.e., as the fiber–matrix adhesion increases, the interphase thickness decreases leading to a lower value of the interphase fraction V_i_/V_m_. In addition, all the above-mentioned variabilities are fiber volume fraction dependent.

### 4.2. Application of HVIM on the Stress Relaxation Results

**Figure 9:**
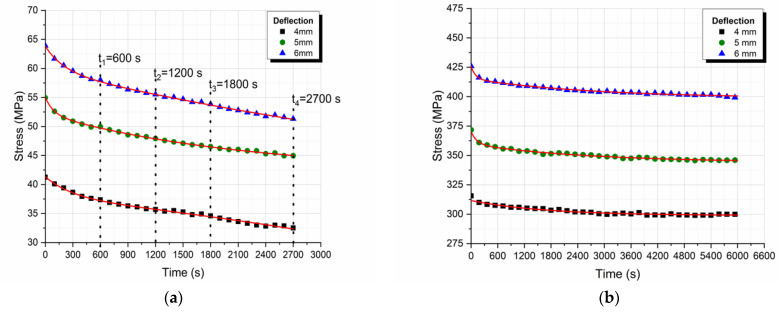
Stress relaxation curves (**a**) of the neat epoxy resin and (**b**) the GFRP composite for different deflection levels.

**Figure 10:**
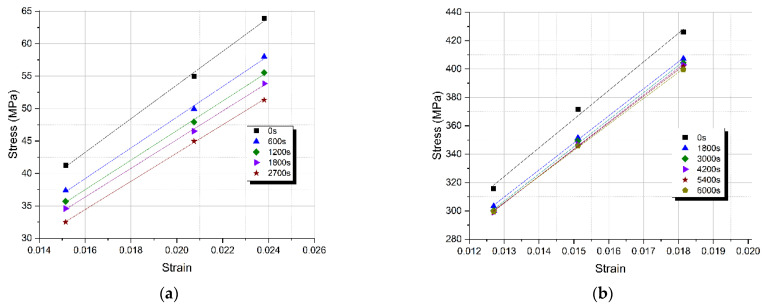
Isochronous curves (**a**) of the neat epoxy resin and (**b**) the GFRP composite at different times.

The stress–time relaxation curves for different levels of deflection are given in Figure 9a,b, for the neat epoxy resin and the GFRP composite respectively. It is observed that as the imposed flexural deflection increases, the initial stress also increases in the various stress relaxation curves, as expected. In both cases, it was observed that stress increases proportionally to the deflection applied. To verify this, isochronous curves were plotted at various times (Figure 10a,b).

The isochronous curves plotted and presented in Figure 10a,b depict a linear viscoelastic behavior, thus it can be deduced that our tests were conducted within the linear viscoelastic region.

**Figure 11:**
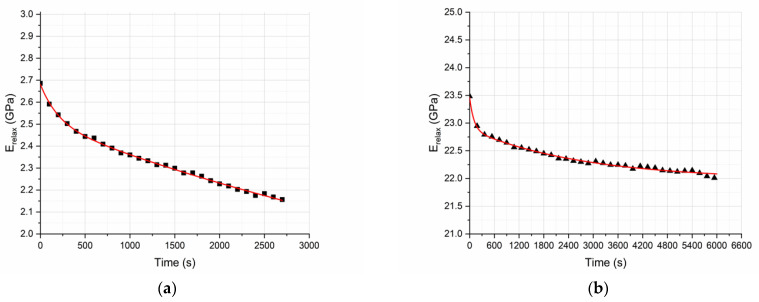
Relaxation modulus of (**a**) the neat epoxy resin and (**b**) the GFRP composite.

Therefore, a single relaxation modulus vs. time curve can be plotted. More precisely taking the slope of the fitted linear isochronous curves, we can plot the relaxation modulus variation with time for both the neat epoxy resin and the GFRP composite (Figure 11a,b).

In Figure 11a,b, the relaxation moduli of both the neat epoxy resin and the GFRP composite are given, as derived by the isochronous curves. It is observed, as anticipated, that the relaxation modulus at t = 0 s corresponds to the quasistatic flexural modulus.

The effect of the glass fibers is clearly visible through the comparison of these two curves (Figure 11a,b). The reduction of the relaxation modulus of GFRP composite specimens over time amounts to 6.8% and is far smaller than the reduction of relaxation modulus of the neat epoxy resin specimens, which amounts to 22.7%. This is attributed to the reduction of the degrees of freedom of the polymeric chains due to their adhesion to the fibers’ surface. As explained thoroughly in Figure 5, due to the existence of the fiber–matrix interphase and the small distance between successive glass fibers, the bulk polymeric matrix in the composite is characterized by modified properties compared to the properties of the neat epoxy resin.

**Figure 12:**
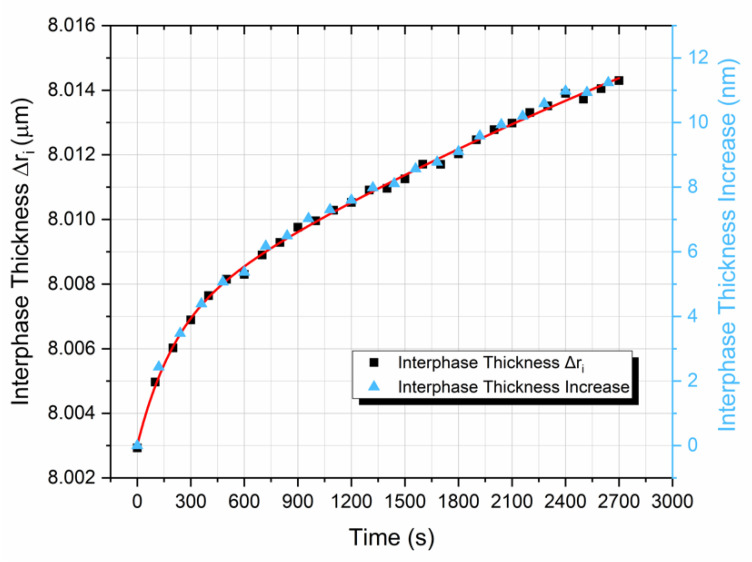
Prediction of the thickness of the hybrid viscoelastic interphase as a function of time.

**Figure 13:**
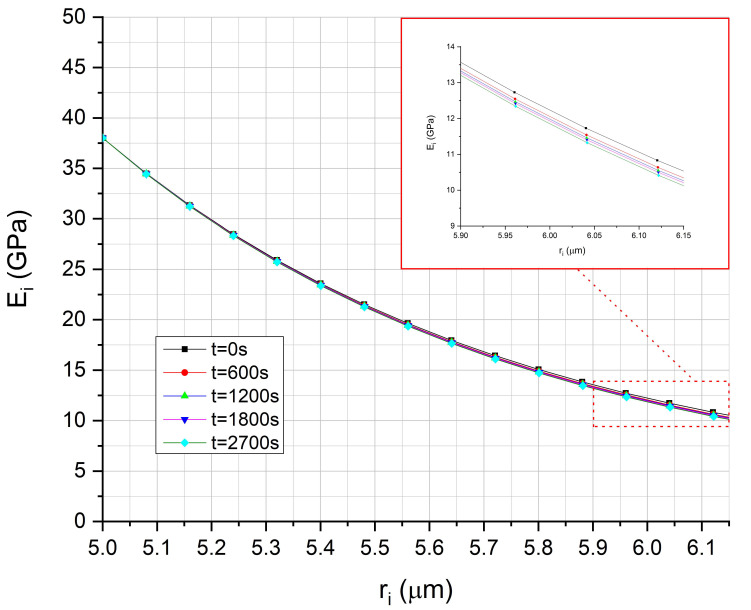
Prediction of hybrid viscoelastic interphase modulus as a function of the distance from the E-glass fiber for different time instants.

Next, by applying the HVIM, as presented in Figure 12, the interphase thickness vs. time variation was deduced. From this figure, one can observe a continuous increase in the interphase thickness with time, during relaxation. Indeed, as stresses relax in the interphase area, the degree of adhesion k_E_ is reduced, and according to the already presented theoretical background, this results in an increase in the interphase thickness. A consequence of the above mechanism is the reduction of the interphase modulus in the radial direction at different time instants during stress relaxation, and this is shown in Figure 13.

## 5. Conclusions

In the present investigation, the hybrid interphase model (HIM) and the hybrid viscoelastic interphase model (HVIM) were presented and subsequently applied to a real GFRP composite subjected to quasistatic three-point bending and three-point bending stress relaxation tests. The whole study was divided into two parts. In the first part, the composite subjected to quasistatic three-point bending tests, and the HIM model dictates that:

- The minimum value (corresponding to the midpoint of the fiber separation distance) of the relative interphase modulus is always higher than the modulus of the neat resin for all fiber volume fractions, while there is a continuous increase of this value with the filler volume fraction V_f_.
- However, when plotting the interphase relative modulus E_i_/E_m_ value corresponding to the midpoint of the fiber separation against adhesion coefficient k_E_, one can observe an initial and almost abrupt increase, reaching a maximum for k_E_ = 0.3, and this is followed by a subsequent reduction of the ratio E_i_/E_m_.
- This kind of behavior was attributed to three different parameters, namely: the interphase thickness, the maximum value of the relative interphase modulus at the fiber–matrix contact point and the rate of decrease of the relative interphase modulus in the radial direction.
- Finally, in the same part of the present investigation, the characteristic surface resulting from the V_i_/V_m_–k_E_–V_f_ three-dimensional diagram was presented.

In the second part, the HVIM model was applied to the same GFRP composite subjected to stress relaxation under three-point bending conditions. It was found that:

- The interphase thickness increases with time during relaxation.
- At any time instant during the stress relaxation process, the interphase modulus always decreases along the radial direction.

Overall, it should be stressed that in the present investigation, a concrete micromechanics model was applied and presented, relating micromechanics interphasial phenomena with the macroscopic mechanical and viscoelastic behavior of a GFRP composite. The whole procedure presented can be applied to any type of fiber–polymer composite.

## Figures and Tables

**Table 1:** Constituents and selected glass fiber-reinforced polymer (GFRP) properties [31].

Property	Matrix	E-Glass	Composite
Density (gcm^−3^)	1.16	2.56	1.7–1.9
Flexural modulus (GPa)	2.5–2.7	76	24
Water absorption (%)	1.16	-	0.15

## Data Availability

Data available on request.
